# TMEM115 is an integral membrane protein of the Golgi complex involved in retrograde transport

**DOI:** 10.1242/jcs.136754

**Published:** 2014-07-01

**Authors:** Yan Shan Ong, Ton Hoai Thi Tran, Natalia V. Gounko, Wanjin Hong

**Affiliations:** 1 Institute of Molecular and Cell Biology, 61 Biopolis Drive, Singapore 138673, Singapore; 2 IMB-IMCB Joint Electron Microscopy Suite, 20 Biopolis Street, Singapore 138671, Singapore; 3 Department of Biochemistry, National University of Singapore, Singapore 117599, Singapore

**Keywords:** TMEM115, Golgi complex, Retrograde transport, COG complex

## Abstract

Searching and evaluating the Human Protein Atlas for transmembrane proteins enabled us to identify an integral membrane protein, TMEM115, that is enriched in the Golgi complex. Biochemical and cell biological analysis suggested that TMEM115 has four candidate transmembrane domains located in the N-terminal region. Both the N- and C-terminal domains are oriented towards the cytoplasm. Immunofluorescence analysis supports that TMEM115 is enriched in the Golgi cisternae. Functionally, TMEM115 knockdown or overexpression delays Brefeldin-A-induced Golgi-to-ER retrograde transport, phenocopying cells with mutations or silencing of the conserved oligomeric Golgi (COG) complex. Co-immunoprecipitation and *in vitro* binding experiments reveals that TMEM115 interacts with the COG complex, and might self-interact to form dimers or oligomers. A short region (residues 206–229) immediately to the C-terminal side of the fourth transmembrane domain is both necessary and sufficient for Golgi targeting. Knockdown of TMEM115 also reduces the binding of the lectins peanut agglutinin (PNA) and *Helix pomatia* agglutinin (HPA), suggesting an altered O-linked glycosylation profile. These results establish that TMEM115 is an integral membrane protein of the Golgi stack regulating Golgi-to-ER retrograde transport and is likely to be part of the machinery of the COG complex.

## INTRODUCTION

The Golgi complex is a central station of the secretory pathway and plays a fundamental role in protein modifications such as glycosylation and protein sorting to multiple post-Golgi compartments (Glick and Nakano, 2009; Miller and Ungar, 2012; Rosnoblet et al., 2013b). The Golgi is dynamically linked to the endoplasmic reticulum (ER) by retrograde Golgi-to-ER transport, which is involved in the retrieval of resident ER proteins (such as luminal KDEL-containing proteins) that have escaped from the ER, as well as maintaining equilibrium of cycling membrane proteins (such as ERGIC53, KDEL receptors, p24 family proteins and SNAREs) in the early secretory pathway (Füllekrug et al., 1999; Tang et al., 1995; Tang and Wang, 2013; Townsley et al., 1993). Retrograde transport from the endosomal compartments to the Golgi also links the endocytic pathway with the secretory pathway (Chia and Gleeson, 2011; Lieu and Gleeson, 2011; Seaman, 2008; Seaman, 2009). Intracellular transport in the secretory and endocytic pathways is tightly regulated and involves the interactions of many organelles and machineries so that cargoes are delivered from one compartment to another while the integrity and the composition of proteins and lipids across these organelles are carefully balanced and maintained (Cancino and Luini, 2013). Both the anterograde and retrograde transport pathways are crucial for the development and maintenance of the normal physiology of an organism. Although it is known that the secretory pathway is important for cell growth, defects in retrograde transport can indirectly result in the failure of the ER-to-Golgi transport pathway. It has been proposed that the defective biosynthetic trafficking observed in some yeast mutants of components of the conserved oligomeric Golgi (COG) tethering complex might be an outcome of secondary trafficking defects arising from primary retrograde trafficking defects (Kim et al., 2001; Wuestehube et al., 1996).

The evolutionarily conserved COG complex has eight subunits (COG1–COG8) and is implicated in the tethering of retrograde Golgi vesicles for intra-Golgi transport as well as retrograde endosome–Golgi transport. Accordingly, malfunctions in the COG complex greatly impact on Golgi integrity, protein and lipid trafficking and glycosylation (Miller and Ungar, 2012; Ungar et al., 2006; Willett et al., 2013). The COG complex is organized into two functionally and structurally distinct sub-complexes, Lobe A (COG1–COG4) and Lobe B (COG5–COG8) (Laufman et al., 2011; Lees et al., 2010; Loh and Hong, 2004; Walter et al., 1998). Subunits of Lobe A are essential for growth in yeast. Deletions or mutations of genes encoding these proteins cause severe glycosylation defects. Conversely, deletions of Lobe B subunits do not exhibit such severe phenotypes in yeast, suggesting that the Lobe B subcomplex has a regulatory or redundant role (Kudlyk et al., 2013; Richardson et al., 2009; Zolov and Lupashin, 2005). It has recently been shown that downregulation of COG function results in the mislocalization and degradation of resident Golgi glycosyltransferases and/or glycosidases (Smith and Lupashin, 2008). The COG complex is intimately involved in glycosylation homeostasis. It maintains a dynamic and finely tuned balance between anterograde and retrograde trafficking by coordinating intra-Golgi retrograde transport. This fine balance is required for the correct localization and distribution of the glycosylation enzymes in the Golgi (Reynders et al., 2011). The functional importance of the COG complex in human is underscored by the identification of mutations in six out of the eight subunits (COG1, COG4, COG5, COG6, COG7 and COG8) in patients with the human disease congenital disorder of glycosylation (CDG) type II (Foulquier et al., 2007; Foulquier et al., 2006; Freeze and Ng, 2011; Kranz et al., 2007; Kudlyk et al., 2013; Ng et al., 2011; Spaapen et al., 2005; Wu et al., 2004; Zeevaert et al., 2008). The patients are characterized by abnormal glycosylation, although the clinical presentations are highly heterogeneous. Cellular depletion of COG subunits or fibroblasts derived from CDG type II patients exhibit resistance to Brefeldin A (BFA)-induced Golgi-to-ER retrograde transport and accelerated degradation of Golgi proteins such as GS15, GS28 mannosidase II, GPP130, CASP, giantin and golgin-84 (Oka et al., 2004; Steet and Kornfeld, 2006). In humans, mutations of both Lobe A and Lobe B subcomplexes are associated with CDG, suggesting that the entire complex is essential for proper function of the Golgi complex to execute normal glycosylation of cargo proteins. Here, we describe a new player in the Golgi-to-ER retrograde transport pathway. TMEM115, also known as PL6, located on chromosome 3p21.3, was originally thought to function as a tumor suppressor (Ivanova et al., 2008). Our biochemical, cell biological and functional studies suggest that TMEM115 is an integral Golgi protein of ∼35 kDa with four predicted transmembrane domains. It interacts with COG4 of the COG complex and is involved in proper O-linked glycosylation and efficient Golgi-ER retrograde transport in response to BFA treatment.

## RESULTS

### Identification of new candidate Golgi membrane proteins

The Human Proteins Atlas database (http://www.proteinatlas.org/) was searched for the term ‘transmembrane protein’. The search was conducted using version 5.0 and produced a list of 3541 proteins, of which 103 had been validated by immunohistological staining. Further examination of the tissue expression pattern and subcellular localization of these 103 proteins on the database narrowed the list of candidate Golgi proteins to 11. In depth examination of the subcellular labeling patterns of these 11 proteins enabled us to focus on five proteins with clear perinuclear Golgi-like labeling (C11orf87, TMEM115, TMEM77, TMEM87A and TMEM17). We used BLAST to align the sequence of these five proteins against proteins in the yeast and fly databases and found that both TMEM115 and TMEM87A have structural homologs in these organisms (the process is outlined in supplementary material Fig. S1A). TMEM115 has been reported to have reduced expression in renal clear cell carcinomas and other VHL-deficient tumors and to exhibit Golgi localization (Ivanova et al., 2008). These observations suggest that it might be a potential tumor suppressor located in the Golgi. Therefore, we focused our study on TMEM115.

Hydrophobicity analysis of the TMEM115 sequence (351 residues) by TMpred (http://www.ch.embnet.org/software/TMPRED_form.html) revealed four hydrophobic regions in the N-terminal region (supplementary material Fig. S1B). Alignment of amino acid sequences of TMEM115 from human, mouse, frog, zebrafish, fly, worm and yeast revealed they are well conserved throughout evolution. The C-terminal region (residues 206–351) is hydrophilic in nature, within which the epitope of the rabbit antibody used in the Atlas study (HPA015497) is located (supplementary material Fig. S1C). Accordingly, TMEM115 was predicted to have four transmembrane domains with both N- and C-termini facing either the cytoplasm (a model is shown in supplementary material Fig. S1C) or the lumen.

### TMEM115 is located at the Golgi complex

**Fig. 1.**
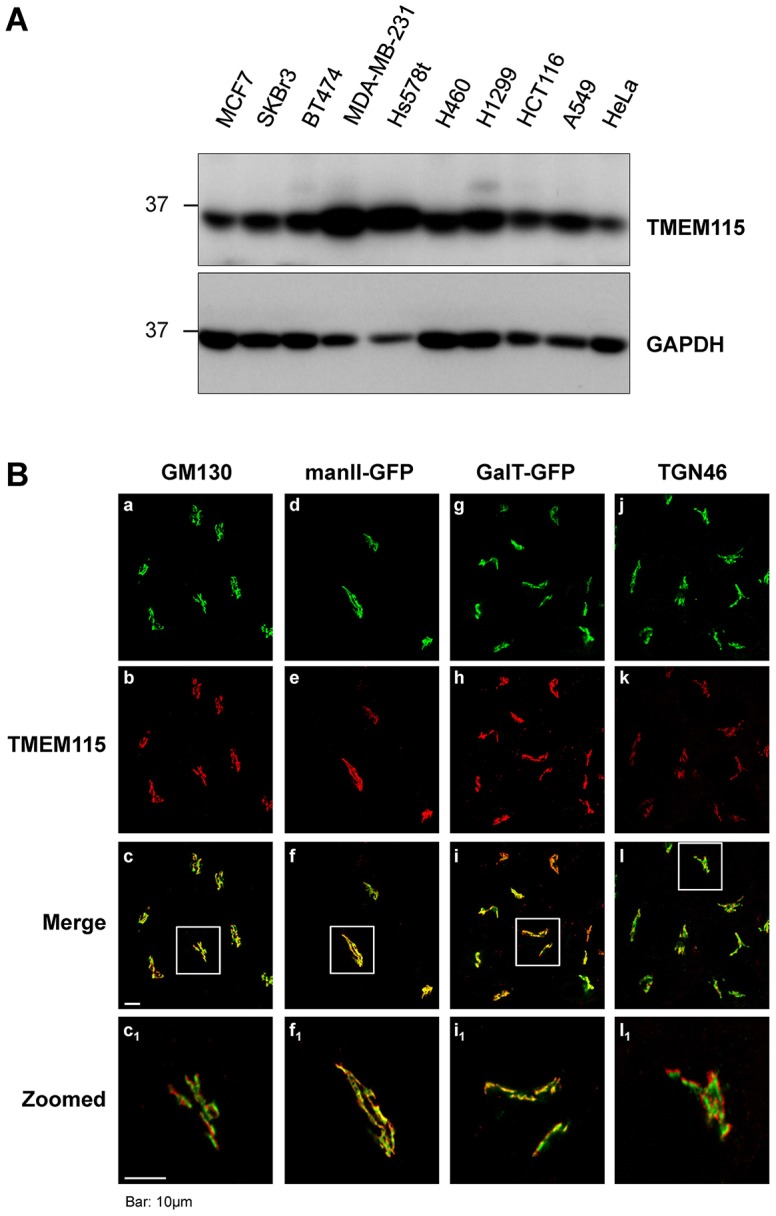
**Endogenous expression and subcellular localization of TMEM115.** (A) Endogenous expression of TMEM115 in various cell lines. (B) Subcellular localization of endogenous TMEM115. Endogenous TMEM115 was colabeled with endogenous GM130 (panels a–c and c_1_) or TGN46 (panels j–l and l_1_) in HeLa cells. In addition, endogenous TMEM115 was colabeled with mannosidase II (manII)–GFP in HeLa cells stably expressing manII–GFP (panels d–f and f_1_), or GalT in HeLa cells stably expressing GalT–GFP (panels g–i and i_1_). Scale bar: 10 µm.

The endogenous levels of TMEM115 in various cell lines were examined by western blotting. As shown in Fig. 1A, TMEM115 is ubiquitously expressed as a protein of ∼35 kDa in the examined cell lines; with the highest expression level in MDA-MB-231 and Hs578t, both of which are invasive breast cancer cells (Kirschmann et al., 1999; Paciotti and Tamarkin, 1988).

The subcellular distribution of TMEM115 was investigated using immunofluorescence microscopy (Fig. 1B). TMEM115/PL6 has previously been shown to exhibit a Golgi localization (Ivanova et al., 2008). In our study, cells were double-labeled with antibody against TMEM115 (Fig. 1B, panels b, e, h and k) and other antibodies against various Golgi markers (Fig. 1B, panels a, d, g and j). TMEM115 was found at the perinuclear region, adjacent to, but not co-localized with the cis-Golgi matrix protein GM130 (Nakamura et al., 1995) (Fig. 1B, panels c and c_1_) or the trans-Golgi network (TGN) marker TGN46 (Ponnambalam et al., 1996; Prescott et al., 1997) (Fig. 1B, panels l and l_1_). In cells stably expressing either GFP-tagged mannosidase II (manII–GFP), a medial-Golgi marker or the GFP–GalT chimera [in which the catalytic domain of β1,4-galactosyltransferase 1 (GalT) is replaced by GFP], a trans-Golgi marker (Colley, 1997; Rabouille et al., 1995; Velasco et al., 1993), TMEM115 was found to be well colocalized with manII–GFP (Fig. 1B, panels f and f_1_) and the signal substantially overlapped with GalT–GFP (Fig. 1B, panels i and i_1_). Immunoelectron microscopy analysis showed that TMEM115 was localized to the Golgi complex and was distributed towards the outer periphery of the Golgi (supplementary material Fig. S2A, arrows). These results indicate that TMEM115 is likely to be enriched in the Golgi stack.

### The C-terminal tail of TMEM115 is exposed in the cytoplasm

**Fig. 2.**
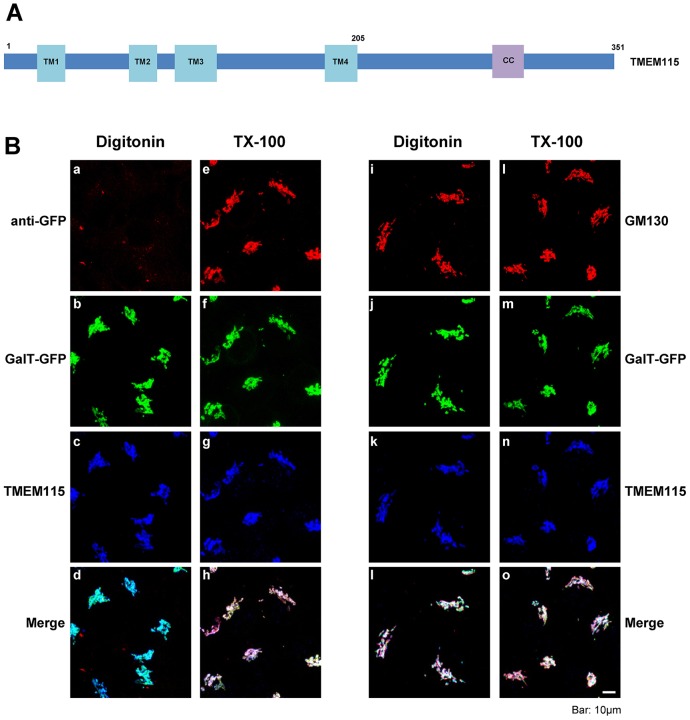
**The C-terminal portion of TMEM115 is exposed in the cytoplasm.** (A) Schematic illustration of the predicted structure of TMEM115. TM, transmembrane domain; CC, coiled-coil domain. (B) The C-terminal part of TMEM115 is exposed in the cytoplasm. HeLa cells stably expressing GalT–GFP cells were permeabilized by 5 µg/ml digitonin (panels a–d, i–l) or 0.1% Triton-X (panels e–h, m–p). Scale bar: 10 µm.

Evolutionary comparison and hydrophobicity analysis of its primary amino acid sequence predict that TMEM115 has a short cytoplasmic domain at the N-terminus, followed by four transmembrane domains and a C-terminal hydrophilic tail of ∼146 amino acid residues (supplementary material Fig. S1C), which contains a predicted coiled-coil domain (Fig. 2A). Immunofluorescence studies in semi-permeabilized cells (in which only the plasma membrane is permeabilized but the membrane of internal organelles such as the Golgi is intact) were carried out to investigate the proposed membrane topology of TMEM115, using an antibody that specifically recognizes an epitope (residues 255–344) located in the C-terminal tail. Low concentrations (5 µg/ml) of digitonin (Diaz and Stahl, 1989; Eckhardt et al., 1999) were used to selectively permeabilize the plasma membrane. The cells were then simultaneously labeled with anti-GFP and anti-TMEM115 antibodies (Fig. 2B, panels a–h), or with anti-GM130 and anti-TMEM115 antibodies (Fig. 2B, panels i–p). As the catalytic domain of GalT is replaced by GFP in the chimera GalT–GFP (Schaub et al., 2006), the GFP tag is oriented to the lumen of the Golgi cisternae and is not accessible to the GFP antibody when the Golgi membrane is not permeabilized. Therefore if the C-terminal tail of TMEM115 is in the lumen of the Golgi, it too cannot be accessed by the TMEM115 antibody. In digitonin-treated cells, there were no significant detection of anti-GFP (Fig. 2B, panels a and d), indicating that the anti-GFP antibody was unable to react with the epitope located in the lumen of an intact Golgi complex. In contrast, the peripheral cytoplasmic protein GM130 (Nakamura et al., 1995) was readily labeled in digitonin-permeabilized cells (Fig. 2B, panel i). Interestingly, TMEM115 was also clearly detected in digitonin-treated cells (Fig. 2B, panels c and k). In the parallel control experiment where cells were fully permeabilized with 0.1% Triton-X 100 (Fig. 2B, panels e–h and m–p), all antibodies were able to bind to their respective epitopes. The intensity of the signals of TMEM115 and GM130 in fully permeabilized cells were not increased as compared with those in digitonin-permeabilized cells (Fig. 2B, panels c and g, k and o, i and m). These results clearly show that anti-TMEM115 antibody was able to fully access its epitope in semi-permeabilized cells, establishing that the C-terminal region of TMEM115 is oriented towards the cytoplasm (supplementary material Fig. S1C).

### Proper expression level of TMEM115 is required for BFA-induced Golgi-to-ER retrograde transport

**Fig. 3.**
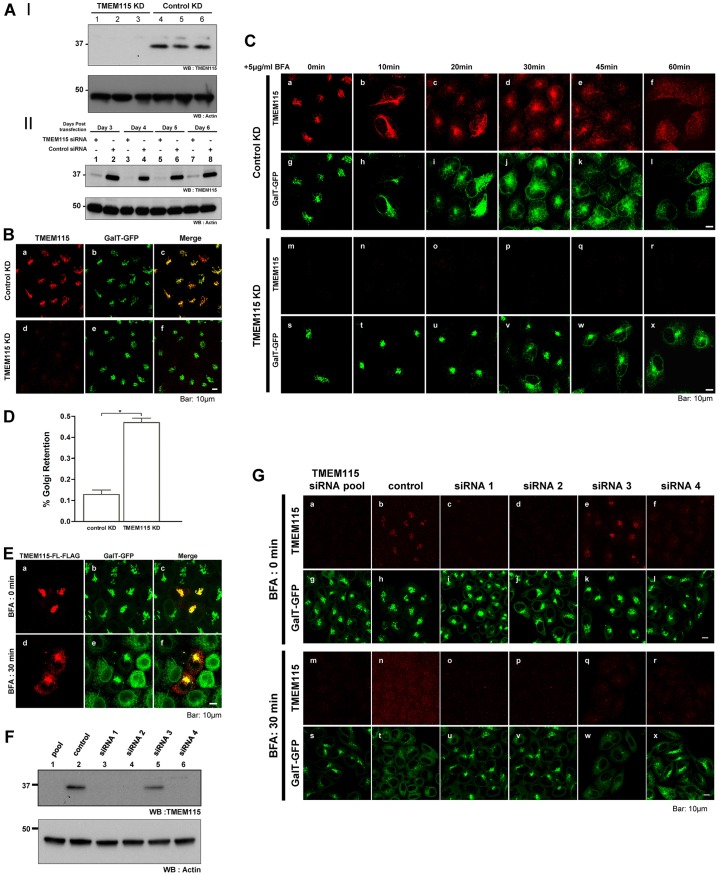
**BFA-induced Golgi disassembly is delayed in both TMEM115-silenced and TMEM115-overexpressing cells.** (A) TMEM115 is effectively silenced using siRNA. (I) Pooled siRNA targeting TMEM115 (lane 1–3) or control pooled non-targeting siRNA (lane 4–6) was transfected into HeLa cells. Cell lysates were harvested 72 hours after transfection. (II) HeLa cells were transfected with pooled siRNA targeting TMEM115 or control pooled non-targeting siRNA. Cell lysates were harvested at the indicated time after transfection. (B) Pooled siRNA targeting TMEM115 (panels d–f) or control pooled non-targeting siRNA (panels a–c) was transfected into HeLa cells stably expressing GalT–GFP. 72 hours after transfection, cells were examined by immunofluorescence microscopy. (C) BFA-induced Golgi disassembly is delayed in TMEM115-silenced cells. HeLa cells stably expressing GalT–GFP were transfected with either pooled siRNA targeting TMEM115 (lower panels) or control pooled non-target siRNA (upper panels). 72 hours after transfection, cells were incubated with 5 µg/ml BFA at 37°C for the indicated length of time. (D) HeLa cells stably expressing GalT–GFP were transfected with control or TMEM115-specific siRNA. The numbers of cells that exhibit Golgi staining after 10 minutes of 5 µg/ml BFA treatment at 37°C were quantified. Control KD, *n* = 1221; TMEM115 KD, *n* = 980. *P<0.0001. (E) BFA-induced Golgi disassembly is delayed in TMEM115-overexpressing cells. C-terminally FLAG-tagged full-length TMEM115 (TMEM115-FL-FLAG) was transfected into HeLa cells stably expressing GalT–GFP. Cells were then incubated with 5 µg/ml BFA at 37°C for 30 minutes. 100% of transfected cells that expressed moderate levels of TMEM115-FL-FLAG exhibited a Golgi retention phenotype after BFA treatment (*n* = 15). (F) Individual siRNAs from the siRNA pool were used to target TMEM115. Pooled siRNA targeting TMEM115 (lane 1) or control pooled non-target siRNA (lane 2) or individual siRNA duplexes targeting TMEM115 (lanes 3–6) were transfected into HeLa cells. Cells were harvested 72 hours after transfection. The lysates were analyzed for the level of TMEM115 by western blotting. (G) BFA-induced Golgi disassembly assay. Pooled siRNA targeting TMEM115 or control pooled non-target siRNA or each individual siRNA duplex targeting TMEM115 was transfected into HeLa cells stably expressing GalT–GFP. Cells were then incubated with 5 µg/ml BFA at 37°C for 0 minutes (upper panels) or 30 minutes (lower panels). Scale bar: 10 µm.

To study the function of TMEM115, we silenced its protein expression using siRNA. Fig. 3A shows (for triplicate samples) that TMEM115 expression was effectively knocked down (Fig. 3A, panel I) and the level of knockdown can be maintained in the cells for at least 6 days post siRNA transfection (Fig. 3A, panel II). Immunofluorescence analysis also showed that the perinuclear staining of TMEM115 was also greatly reduced (Fig. 3B, panel d).

We then examined the effect of TMEM115 depletion on various Golgi-associated functions [such as the Golgi morphology, vesicular stomatitis virus G protein (VSV-G) transport and BFA-induced Golgi-to-ER transport] and found that the Golgi complex became more compact (Fig. 3B, panel e) and that BFA-induced Golgi disassembly was affected (Fig. 3C). VSV-G protein transport in TMEM115-silenced cells did not exhibit any difference to the control cells (data not shown). To follow BFA-induced Golgi-to-ER retrograde transport, control and TMEM115-silenced cells were incubated with 5 µg/ml BFA at 37°C for various times. The redistribution of TMEM115 and the Golgi marker GalT–GFP were visualized by immunofluorescence microscopy (Fig. 3C). In control cells (Fig. 3C, top panels), both TMEM115 and GalT–GFP were found in tubule-like structures spreading out from the perinuclear Golgi region after incubation with BFA for 10 minutes. By 30 minutes, the majority of GalT–GFP had been redistributed into an ER-like labeling pattern, whereas TMEM115 was found in both the perinuclear region and diffuse punctated structures. By 45–60 minutes, TMEM115 had been redistributed in punctated structures scattered throughout the cytoplasm. In marked contrast, GalT–GFP was found in a tight, compact structure at the perinuclear region in TMEM115 knocked-down cells 10–20 minutes after treatment with BFA. This labeling pattern of GalT–GFP persisted even after 30 minutes of incubation with BFA, after which the protein gradually redistributed to an ER-like pattern (at 45 and 60 minutes). Even after 45–60 minutes incubation, GalT–GFP had not completely re-distributed back to the ER (Fig. 3C, bottom panels). The quantification of these experiments was shown in Fig. 3D. Similar results were also observed in other cell lines, such as A549, H460 using endogenous Golgi proteins such as giantin and GPP130 (data not shown), suggesting that silencing of TMEM115 markedly delays BFA-induced Golgi-to-ER retrograde transport.

The BFA-dependent Golgi disassembly was also examined in cells transiently overexpressing exogenous full-length TMEM115 (TMEM115-FL). Fig. 3E shows that transiently expressed TMEM115-FL was located to the Golgi complex and colocalized with GalT–GFP (Fig. 3E, panels a–c). Interestingly, when cells were treated with BFA for 30 minutes, both GalT–GFP and TMEM115-FL were found in a compact structure at the perinuclear region, whereas in the surrounding untransfected cells, GalT–GFP was efficiently redistributed to the ER (Fig. 3E, panels d–f). This delay of BFA-induced Golgi disassembly is similar to that in TMEM115 knocked-down cells. The strength of inhibition of BFA induced Golgi redistribution also correlates with the level of expression of this construct [i.e. the higher the expression of TMEM115-FL, the slower the rate of BFA-induced Golgi redistribution (supplementary material Fig. S3A, panels a and b, asterisks)]. These observations suggest that both overexpressing and silencing TMEM115 inhibits BFA-induced Golgi disassembly; therefore, BFA-induced Golgi-to-ER retrograde transport might require finely regulated levels of TMEM115.

To rule out any possibility of off-target effects in the siRNA experiments, we proceeded to validate the effect of TMEM115 silencing in cells by using specific siRNAs from the siRNA pool against TMEM115. Immunoblotting analysis results showed that, of the four siRNAs, siRNA 3 had the least efficiency in silencing TMEM115 (Fig. 3F, Fig. 3G, panel e). It also did not affect the redistribution of GalT–GFP to the ER in the presence of BFA, similar to control cells (Fig. 3F, panel w). By contrast, the other three siRNA duplexes were able to knockdown TMEM115 efficiently, and also had an inhibitory effect on GalT–GFP redistribution to the ER, similar to that of the pooled TMEM115 siRNA (Fig. 3G). In addition, the Golgi complex (marked by GalT–GFP) appeared to be more compact in TMEM115-silenced cells as compared to the control cells. Similar to the Golgi disassembly assay by BFA, the degree of compactness correlates to the level of TMEM115 silencing. siRNA 3, which has the least efficiency for TMEM115 silencing, shows the least compact in Golgi morphology (Fig. 3G; supplementary material Fig. S3B).

Silent mutations were introduced into TMEM115–FLAG to make it resistant to one (siRNA 1) of the siRNAs used in our experiments. Expression of TMEM115 in the knockdown cells could be rescued by the exogenous siRNA-resistant construct (TMEM115 si#1R-FLAG) as shown by immunofluorescence (supplementary material Fig. S4A, panels i and l) and western blotting (supplementary material Fig. S4B). Similar to wild type, TMEM115 si#1R-FLAG, when expressed at very high levels, disrupts the Golgi complex (supplementary material Fig. S4A, panel a,b, panel i–j, #). In cells that highly expressed TMEM115 si#1R-FLAG (supplementary material Fig. S4A, cells marked with #), the entire Golgi (labeled by GalT–GFP) was dispersed throughout the cell. However, when expressed at a moderate level, siRNA-resistant TMEM115 was located at the Golgi complex and colocalized with GalT–GFP (supplementary material Fig. S4A, panels a–d; panels i–l, arrowheads). In control cells, upon 10 minutes treatment with BFA, both the FLAG-tagged protein and GalT–GFP were observed in a compact structure at the perinuclear region in 91% of the cells that were moderately expressing TMEM115 si#1R-FLAG, whereas in the surrounding untransfected cells, GalT–GFP was efficiently redistributed to the ER (supplementary material Fig. S4A, panel f). This delay effect on the BFA-induced Golgi-to-ER retrograde transport is similar to that caused by the silencing of TMEM115 (supplementary material Fig. S4A, panel n, arrow). Therefore, it seems that the BFA-dependent Golgi-to-ER retrograde transport is regulated by a finely balanced amount of TMEM115. The expression of TMEM115 si#1R-FLAG in TMEM115-silenced cells appears to be able to rescue this delayed phenotype in some (supplementary material Fig. S4A, panels m and n, asterisks), but not all cells expressing the FLAG-tagged TMEM115. However, we are unable to differentiate whether this reversion to a wild-type phenotype is due to a true rescue event or due to the fact that the Golgi was already disrupted by a high level expression of TMEM115 si#1R-FLAG.

The effect of TMEM115 depletion on BFA-dependent Golgi disassembly is similar to the phenotype observed in cells deficient in COG proteins (Kranz et al., 2007; Steet and Kornfeld, 2006) or upon silencing COG subunits (Laufman et al., 2011; Laufman et al., 2013; Laufman et al., 2009), indicating that TMEM115 might play certain functions in COG-dependent transport pathways. Taken together, these results strongly suggest that TMEM115 functions in regulating or directly involved in the retrograde transport from the Golgi to the ER.

### TMEM115-FL interacts with β-COP, COG proteins and ERGIC53

**Fig. 4.**
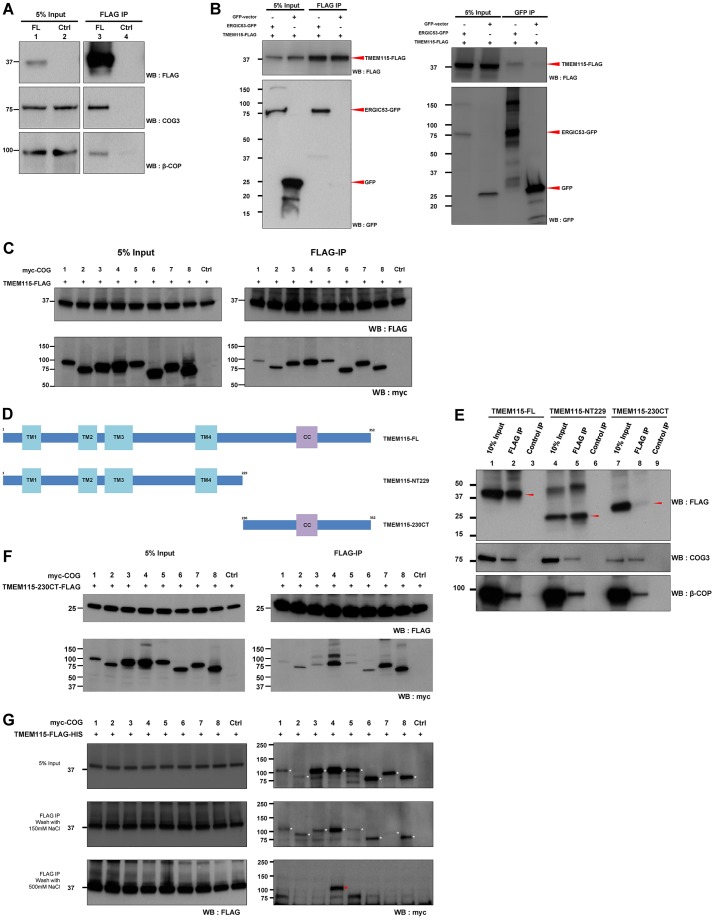
**TMEM115 associates with proteins involved in retrograde trafficking.** (A) TMEM115-FL-FLAG interacts with endogenous COG3 and β-COP. TMEM115-FL-FLAG was transfected into HEK293 cells. Lysates derived from control cells (Ctrl, lanes 2 and 4) or transfected cells (FL, lanes 1 and 3) were subjected to FLAG immunoprecipitation (IP). (B) TMEM115-FL–FLAG interacts with ERGIC53–GFP. HEK293 cells were transfected with TMEM115-FL–FLAG and either GFP empty vector or ERGIC53–GFP. Cell lysates were subjected to FLAG or GFP immunoprecipitation. (C) TMEM115 interacts with all COG subunits. HEK293 cells were transfected with TMEM115-FL–FLAG and either empty vector or each of Myc–COG proteins. Cell lysates were subjected to FLAG immunoprecipitation. (D) Schematic illustration of the FLAG-tagged TMEM115 full-length and mutants. All proteins are C-terminally tagged with FLAG. TMEM115-FL, full-length TMEM115; TMEM115-NT229 contains residues 1–229 of TMEM115; TMEM115-230CT contains residues 230–252. TM, transmembrane domain; CC, coiled-coil domain. (E) TMEM115 can co-immunoprecipitate COG3 and β-COP through its N- and C-terminal portion. HEK293 cells were transfected with TMEM115-FL, TMEM115-NT229 or TMEM115-230CT. Cell lysates were subjected to FLAG immunoprecipitation. The immunoprecipitates were analyzed by immunoblotting as indicated. The red arrowheads indicate the respective FLAG-tagged TMEM115 proteins. (F) The C-terminal tail of TMEM115 interacts with all COG subunits. HEK293 cells were transfected with TMEM115-230CT and either empty vector or each of Myc–COG proteins. Cell lysates were subjected to FLAG immunoprecipitation. (G) TMEM115 binds to COG4 with the highest affinity. TMEM115 and the eight COG subunits were individually translated *in vitro*. Each of the translated COG proteins was then added to the translated full-length TMEM115–FLAG–HIS and subjected to FLAG immunoprecipitation. The beads were washed with cell lysis buffer containing either 150 mM or 500 mM NaCl. The immunoprecipitates were analyzed by immunoblotting as indicated. The white asterisks indicate respective *in vitro* translated Myc-tagged COG proteins. The red asterisks indicate Myc-tagged COG4.

To understand the function of TMEM115 in Golgi-to-ER transport, we investigated its potential interacting partners, using co-immunoprecipitation and immunoblotting (Fig. 4). Fig. 4A showed that FLAG-tagged TMEM115-FL was efficiently immunoprecipitated and both endogenous proteins of β-COP and COG3 were specifically co-immunoprecipitated. TMEM115-FL was able to co-immunoprecipitate ERGIC53–GFP and vice versa (Fig. 4B). Additional negative controls for TMEM115 interactions are shown in supplementary material Fig. S2C. p230 (also known as GOLGA4) and golgin-84 (also known as GOLGA5) were not co-immunoprecipitated by TMEM115. p230 is a Golgi peripheral protein (Kjer-Nielsen et al., 1999), whereas golgin-84 is a single-transmembrane Golgi integral protein (Bascom et al., 1999). Taken together, these data indicate that the interactions of TMEM115 and its various interacting partners (ERGIC53, β-COP and COG3) are specific.

We further examined whether TMEM115-FL also interacts with other subunits of the COG complex (Fig. 4C). The results demonstrated that all eight COG subunits were each co-immunoprecipitated with TMEM115-FL, with varying efficiencies. To investigate which domains of TMEM115 interact with COG proteins, we generated two C-terminally FLAG-tagged deletion mutants (Fig. 4D). TMEM115-NT229 consists of the first 229 residues of TMEM115, thus containing all the four candidate transmembrane domains. TMEM115-230CT contains the C-terminal tail (residues 230–351). Fig. 4E shows that both TMEM115-NT229 and TMEM115-230CT were able to co-immunoprecipitate endogenous COG3 and β-COP. TMEM115-230CT was also able to co-immunoprecipitate all eight transfected Myc-tagged COG proteins (Fig. 4F).

We further investigated the interactions between TMEM115 and the COG complex by *in vitro* binding assays. TMEM115 and the eight COG subunits were individually translated *in vitro*. Each of the translated COG proteins were then added with the translated full-length TMEM115–FLAG–HIS and subjected to FLAG immunuoprecipitation (Fig. 4G). The middle panel shows that at normal salt washing concentration (150 mM NaCl); TMEM115 was able to co-immunoprecipitate all eight COG subunits albeit with different efficiencies. However, when subjected to a higher salt washing concentration (500 mM NaCl), only COG4 was co-immunoprecipitated by TMEM115 (Fig. 4G, lower panel, red asterisk), indicating that TMEM115 has the highest affinity to COG4. These results suggest that TMEM115 is an interacting partner of the COG tethering complex and its interaction with the complex is likely through direct interaction with COG4, although additional experiments are needed to validate this.

### TMEM115 self-interacts through its N-terminal portion

**Fig. 5.**
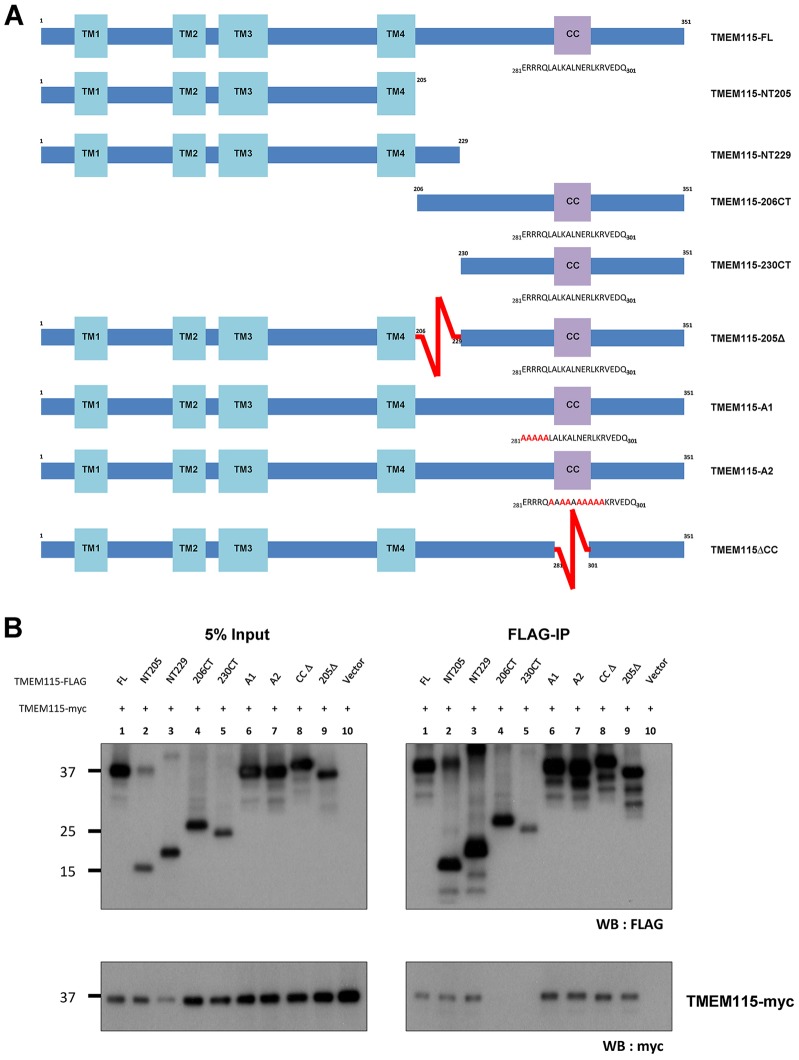
**TMEM115 self-interacts via the N-terminal region.** (A) Schematic illustration of TMEM115 full-length and the various mutants. All proteins were C-terminally tagged with FLAG. TMEM115-FL, full-length TMEM115; TMEM115-NT229 contains residues 1–229 of TMEM115; TMEM115-230CT contains residues 230–252. TMEM115-NT205 contains residues 1–205; TMEM115-206CT has residues 206–252. Residues 206–229 were deleted in TMEM115-205Δ construct. The coiled-coil domain, residues 281–301, was deleted in TMEM115-ΔCC. Residues 281–285 (ERRRQ) was replaced by alanine residues in TMEM115-A1; residues 286–295 (LALKALNERL) was replaced by alanine residues in TMEM115-A2. TM, transmembrane domain; CC, coiled-coil domain. (B) TMEM115 forms homo-oligomers through its N-terminal portion. HEK293 cells were transfected with C-terminally Myc-tagged full-length TMEM115 (TMEM115-myc) and either empty vector or FLAG–TMEM115 mutants. Cell lysates were subjected to FLAG immunoprecipitation (IP). The immunoprecipitates were analyzed by immunoblotting as indicated. All mutants containing the intact N-terminal 205 residues displayed interaction with TMEM115-FL; whereas the two C-terminal fragments lacking the transmembrane regions showed no interaction.

Given that TMEM115 contains four candidate transmembrane domains and a coiled-coil domain, we speculated that the protein might be able to dimerize or oligomerize. To examine whether TMEM115 forms oligomers, we generated six additional deletion mutants of the protein. A graphical representation of all the deletion mutants is shown in Fig. 5A. The ability of each deletion mutant to co-immunoprecipitate the C-terminally Myc-tagged full-length TMEM115 (TMEM115–Myc) was accessed. Fig. 5B shows that TMEM115–Myc was co-immunoprecipitated with FLAG-tagged full-length TMEM115, as well as with most of the TMEM115 mutants, indicating that TMEM115 can form dimers or homo-oligomers. However, TMEM115–Myc was not found to interact with the C-terminal tail of TMEM115 (TMEM15-206CT and TMEM115-230CT). These results indicate that TMEM115 forms homo-dimer or -oligomers through the N-terminal region.

### The C-terminal tail of TMEM115 contains a Golgi-targeting signal within the region of residues 206–229

**Fig. 6.**
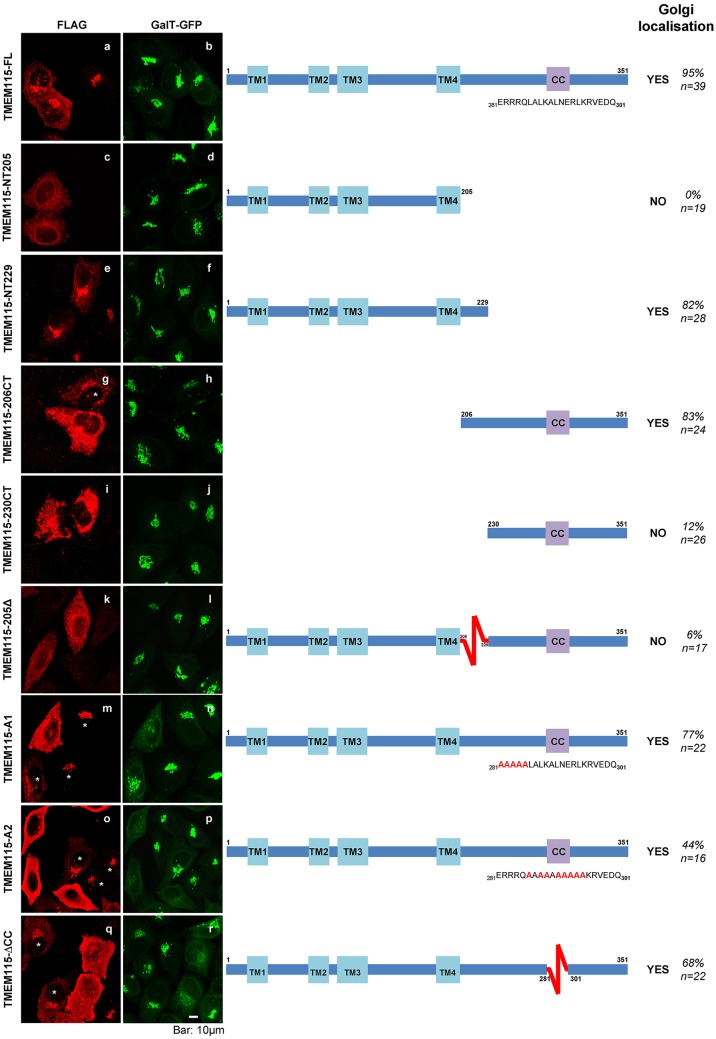
**Residues 206–229 of TMEM115 contain a Golgi-targeting signal.** FLAG-tagged TMEM115 and its mutants were expressed in transfected HeLa cells stably expressing GalT–GFP cells and the localization of the mutants was shown together with GalT–GFP. The percentage of cells expressing Golgi localization for each mutant is also shown. *n*, number of cells counted. TM, transmembrane domain; CC, coiled-coil domain. Scale bar: 10 µm.

To determine which region of TMEM115 is important for Golgi targeting, the subcellular localization of the various mutants were examined. We focused our analysis on cells expressing moderate levels of exogenous proteins. Exogenously expressed full-length TMEM115 exhibited a perinuclear staining (Fig. 6, panel a) which colocalized with GalT–GFP, similar to that of the endogenous protein (Fig. 3B). Mutants harboring mutations in the coiled-coil domain (TMEM115-ΔCC, TMEM115-A1 and TMEM115-A2) did not affect the Golgi localization of TMEM115 (Fig. 6, panels m, o and q, asterisks), indicating that the coiled-coil domain does not directly participate in the targeting the protein to the Golgi. TMEM115-NT205, which contains the four predicted transmembrane domains, would be expected to be localized to the membrane, whereas the TMEM115-206CT fragment would be predicted to be expressed as a cytosolic protein. Surprisingly, TMEM115-NT205 showed an ER-like staining, whereas TMEM115-CT206 exhibited some Golgi staining which partially colocalized with GalT–GFP (Fig. 6, panel g, asterisk). These results suggest that the Golgi localization signal is contained between the end of the fourth transmembrane domain (amino acid 206) and the start of the coiled-coil domain (amino acid 281) of TMEM115. TMEM115-NT229 (a longer version of TMEM115-NT205) was observed at the Golgi complex, whereas the reciprocate shorter C-terminal mutant TMEM115-230CT did not show the Golgi staining. These results suggest that residues 206–229 might contain an ER export and/or Golgi-targeting signal. This conclusion is further supported by the finding that TMEM115-205Δ, which lacks only residues 206–229, did not localize to the Golgi but exhibited an ER-like staining (Fig. 6, panel k).

**Fig. 7.**
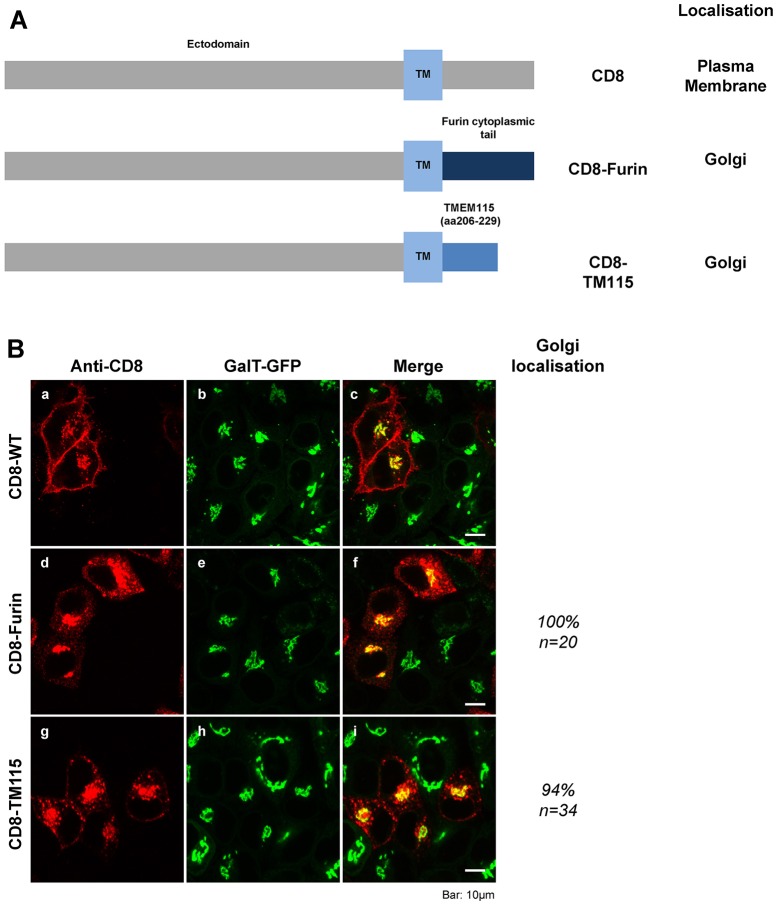
**Residues 206–229 of TMEM115 are sufficient for Golgi targeting.** (A) Schematic illustration of various chimeric CD8 proteins. CD8, full-length wild-type human CD8; CD8–furin, the cytoplasmic tail of CD8 was replaced with the cytoplasmic tail of furin; CD8–TM115, the cytoplasmic tail of CD8 was replaced with the fragment 206–229 of TMEM115. TM, transmembrane domain. (B) Steady state distribution of CD8 or CD8 chimeras in HeLa cells. These proteins were transfected into HeLa cells stably expressing GalT–GFP cells. The chimeras were visualized by staining with anti-CD8 antibody. The percentage of cells expressing Golgi localization for each chimera is also shown. *n*, number of cells counted. Scale bar: 10 µm.

To further validate the role of residues 206–229, we generated a CD8 reporter protein, in which the cytoplasmic tail of CD8 was replaced with the fragment 206–229 of TMEM115 (Fig. 7A). This chimera is called CD8–TM115. This approach has been used previously to functionally test the targeting function of cytoplasmic tails (Seaman, 2004). CD8 is a cell surface protein that is not normally expressed in HeLa cells and it does not appear to have intrinsic targeting information in the intracellular organelles. In CD8–furin, the cytoplasmic tail of CD8 is replaced by that of furin. The cytoplasmic tail of furin has previously been shown to mediate the targeting of the CD8–furin chimera to the Golgi (Hirst et al., 2005). Therefore, if residues 206–229 of TMEM115 functions only as an ER export signal, the chimera CD8–TM115 would be expected to target to the plasma membrane, whereas, if it contains a Golgi-targeting signal, then the chimera would be retained in the Golgi.

Wild-type CD8 was targeted to the plasma membrane (Fig. 7B, panel a), whereas CD8­–furin chimera was found at the Golgi, colocalized with GalT–GFP (Fig. 7B, panel d). CD8–TM115 was also found to colocalize with GalT–GFP at the perinuclear region. Additionally, there are some CD8–TM115 signals at a lower density found at the plasma membrane and punctated structures (Fig. 7B, panel g). These results suggest that the fragment 206–229 of TMEM115 contains a signal that is necessary and sufficient for Golgi targeting.

### Silencing TMEM115 affects glycosylation

**Fig. 8.**
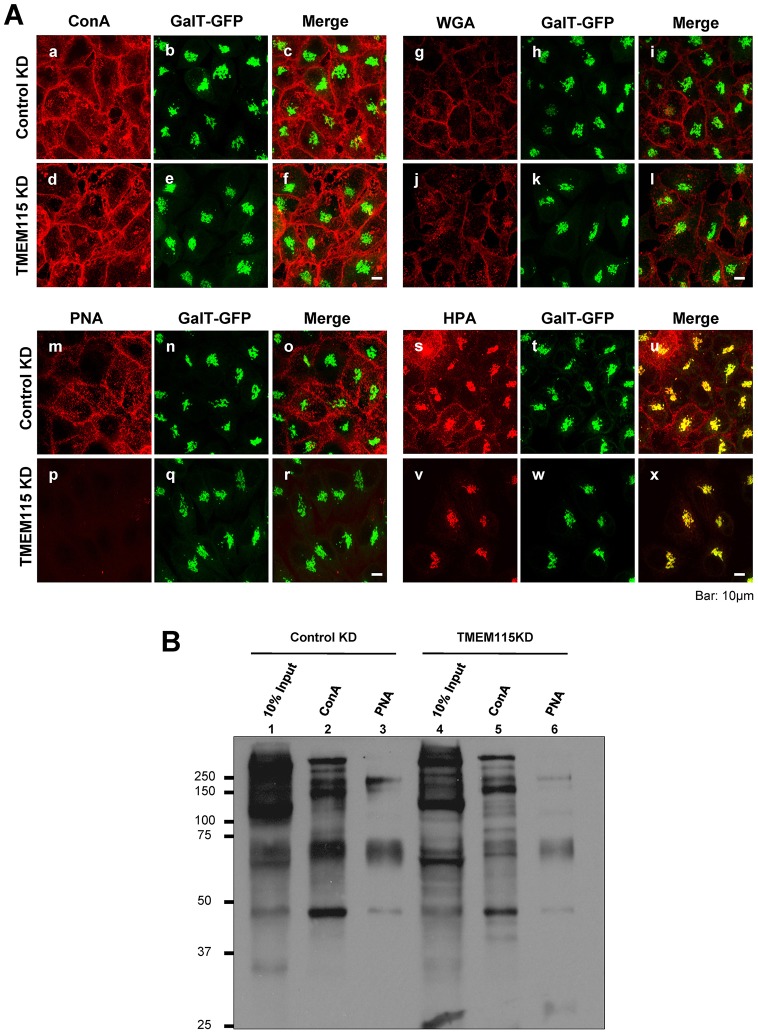
**Gene silencing of TMEM115 affects glycosylation.** (A) Glycosylation is affected in TMEM115 knocked-down cells. HeLa cells stably expressing GalT–GFP were transfected with either pooled siRNA targeting TMEM115 (right panels) or control pooled non-target siRNA (left panels). Non-permeabilized cells were labeled with fluorescent-dye-conjugated lectins. HPA mainly stains the Golgi complex (Campadelli et al., 1993; Virtanen, 1990) with very weak staining at the cell surface (de Albuquerque Garcia Redondo et al., 2004). Therefore, for proper visualization of HPA labeling, the cells were permeabilized with 0.1% Triton-X-100 prior to staining. Scale bar: 10 µm. (B) HeLa cells were transfected with either control non-target siRNA or TMEM115 siRNA. 72 hours after transfection, cells were surface-biotinylated and harvested. Cell lysates were incubated with streptavidin–agarose beads and biotinylated proteins were then eluted from streptavidin–agarose beads and incubated with either Concanavalin A (ConA)–agarose beads or PNA–agarose beads. Proteins were resolved by SDS-PAGE and visualized by western blotting using streptavidin–HRP.

It has been reported that glycosylation is affected in COG-deficient cells (Kranz et al., 2007; Reynders et al., 2011; Rosnoblet et al., 2013b; Zeevaert et al., 2008). Given that TMEM115 interacts with COG proteins and its knockdown also phenocopies COG mutant cells in BFA-induced Golgi-to-ER retrograde transport (Kranz et al., 2007; Laufman et al., 2011; Laufman et al., 2013; Laufman et al., 2009; Steet and Kornfeld, 2006), we proceeded to investigate whether glycosylation was also affected in TMEM115 knocked-down cells. TMEM115-silenced cells were fixed and labeled with fluorescent-dye-conjugated lectins (Fig. 8A). The immunofluorescence results showed that there were no obvious difference in the intensity and labeling pattern of Concanavalin A (ConA) or wheat germ agglutinin (WGA). Surface, but not Golgi, labeling by *Helix pomatia* agglutinin (HPA) was reduced, whereas surface labeling by peanut agglutinin (PNA) was almost completely ablated in the TMEM115 knocked-down cells. We also compared the cell surface biotinylation profile between TMEM115-silenced cells and control cells using surface biotinylation, lectin-binding and immunoblotting analysis (Fig. 8B). Total glycosylation appeared to be reduced in the knockdown cells. ConA-binding glycoproteins were decreased, albeit to a lesser extent as compared to that of PNA binding. ConA, WGA and HPA binds N-linked glycans (Molin et al., 1986; Nagata and Burger, 1974; Sharon, 1983; Sheldon et al., 1998), whereas PNA binds to O-linked glycans (Lotan et al., 1975). However, it has been recently shown that HPA was also capable of recognizing O-linked *N*-acetylglucosamine (O-GlcNAc)-containing glycoproteins (Rambaruth et al., 2012). These results suggest that the function of TMEM115, either directly or indirectly, is required for proper glycosylation to occur within the cell, especially for O-linked glycosylation.

## DISCUSSION

TMEM115 is a multi-transmembrane domain protein, which is located at the Golgi complex (Ivanova et al., 2008). In this study, we found that TMEM115 is likely localized at the medial- and trans-Golgi cisternae and that the protein is oriented in such a way that the C-terminal tail is exposed in the cytoplasm. Moreover, silencing of TMEM115 using siRNA leads to a delay in Golgi-to-ER retrograde transport in the presence of BFA, suggesting that TMEM115 might play a role in regulating this trafficking pathway. Surprisingly, transient overexpression of exogenous TMEM115 also causes a similar delay, indicating that a finely maintained expression level of TMEM115 is required for efficient BFA-sensitive Golgi-to-ER retrograde transport. Exogenously expressed TMEM115 was able to co-immunoprecipitate both β-COP and the COG complex, two of the key players regulating Golgi-to-ER retrograde trafficking pathway (Oka et al., 2004), suggesting that TMEM115 might participate in the regulation of the COPI- and COG-dependent Golgi-to-ER retrograde trafficking pathway.

A series of deletion mutants was created to study the functional domains of TMEM115. Interestingly, the mutant TMEM115-206CT, which does not contain any transmembrane domain and thus was expected to be expressed as a cytosolic protein, was found to target to the Golgi complex. Further investigation showed that when residues 206–229 were removed, the mutant lost its Golgi localization. These 24 amino acids, when added back to the ER-localized mutant (TMEM115-NT205), were able to target the longer version (TMEM115-NT229) to the Golgi complex. When the cytosolic tail of the plasma membrane protein CD8 was replaced with residues 206–229 of TMEM115, the resulting chimera CD8–TM115 was targeted to the Golgi complex instead of the plasma membrane, indicating that an autonomous Golgi-targeting signal is located within these 24 residues.

The TMEM115 expression profile in cancer cell lines shows that TMEM115 seems to be expressed at a higher level in invasive breast cancer cells, such as MDA-MB-231 and Hs578T; as compared to MCF7, a weakly invasive breast cancer cell line. When TMEM115 expression was reduced in MDA-MB-231 cells using siRNA, a substantial inhibitory effect on the growth of these cells was observed (data not shown). These results suggest that there might be an association between elevated TMEM115 expression level and cancer cell properties. However, by contrast, it has been reported that there is a loss of TMEM115 expression in renal clear cell carcinomas and other VHL-deficient tumors (Ivanova et al., 2008). The reason for this discrepancy is not clear, but it is possible that it is specific to the cell type. Future studies will be necessary to explore these possibilities.

Both the N-terminal transmembrane-containing mutant (TMEM115-NT229) and the cytosolic domain mutant (TMEM115-230CT) were able to interact with β-COP and COG3. Given that TMEM115 seems to be able to self-oligomerize through its N-terminal domain (Fig. 5B), it is likely that NT229 might be able to form a complex with wild-type TMEM115 and thus be able to pull-down COG3 and β-COP. Interestingly, even though the cytosolic 230CT does not interact with wild-type TMEM115 (Fig. 5B), it was still able to pull-down COG3 and β-COP. This indicates that TMEM115 might interact with COG3 and β-COP through the C-terminal domain. We have also shown that TMEM115 binds to COG4 with the highest affinity amongst the subunits of the COG complex. Therefore it is likely that associations of TMEM115 with the other COG subunits and the proteins involved in the retrograde transport pathway might be through its interactions with COG4.

Glycosylation appears to be defective in TMEM115-silenced cells, especially the glycosylation of PNA-binding glycans. PNA binds to terminal Gal-β1-3 acetylgalactosamine, a common O-linked glycoprotein motif (Maupin et al., 2012; Sheikh et al., 1999). The lack of PNA staining on TMEM115-silenced cells suggests that the presence of O-linked glycoproteins on the cell surface was greatly reduced. O-linked glycans have been implicated in multiple and diverse biological functions (Brockhausen, 2006; Hang and Bertozzi, 2005; Tarp and Clausen, 2008; Tian and Ten Hagen, 2009). O-glycosylation can substantially increase the hydrophilicity and turgidity of cell surface glycoproteins and can affect the adhesive properties of cells or create binding sites for glycoproteins (Hang and Bertozzi, 2005; Ley and Kansas, 2004). There have been numerous reports regarding the association between cancerous transformations and an increase in short O-glycans, perhaps because disturbance in cell adhesion could facilitate cell migration and metastasis (Brockhausen, 2006; Itzkowitz et al., 1990; Nakamori et al., 1993; Ogata et al., 1998). Changes in the cell surface glycosylation profile are often associated with malignant transformation in cancer (Hakomori, 2002; Hakomori and Cummings, 2012; Ono and Hakomori, 2004). Regulation of O-glycosylation is thought to depend mostly on the expression levels of the initiating GalNAc-T enzyme. Recently, it has also been suggested that the COPI-dependent Golgi-to-ER relocation of initiation GalNac-T enzyme provides another mode of regulation for this glycosylation process (Gill et al., 2010; Gill et al., 2011).

Recently, TMEM165, a novel six transmembrane Golgi protein with no known biological function to date, has been identified to be involved in CDG type II. Mutations in TMEM165 were observed in patients with Golgi glycosylation defects. These mutations crucially changed the subcellular localization of the protein, suggesting that the glycosylation defects observed might be secondary, and that the primary defect could exist outside the glycosylation machinery. Zeevaert and colleagues proposed that TMEM165 is a member of the ‘CDG plus’ subgroup. This group encompass CDG that results from deficiencies of proteins that are not specifically involved in glycosylation but that have functions in the organization and homeostasis of the intracellular compartments and the secretory pathway (Foulquier, 2009; Foulquier et al., 2012; Rosnoblet et al., 2013a; Zeevaert et al., 2013), such as the COG proteins (Foulquier, 2009), SEC23B (Bianchi et al., 2009) and ATP6V0A2 (Guillard et al., 2009).

In summary, this study shows that TMEM115 contains several functional domains: the N-terminal domain, which has several transmembrane domains and mediates the formation of TMEM115 homo-oligomers; residues 206–229, which contain an autonomous Golgi-targeting signal; and its cytosolic C-terminal tail, which mediates interaction with the cytosolic proteins of the retrograde transport machinery, such as the coat complex COPI and the tethering factor COG complex. O-linked glycosylation was shown to be greatly reduced in TMEM115 knocked down cells, although the mechanism by which TMEM115 silencing alters O-glycosylation has yet to be defined. One could speculate that because TMEM115 participates in the regulation of Golgi-to-ER retrograde transport, a defect in this trafficking pathway would thereby in turn affect the distribution and/or localization of glycosylation enzymes that are necessary for effective glycosylation. Therefore, TMEM115 might be a new member of the CDG subgroup ‘CDG plus’, and future analysis of human patients displaying a CDG phenotype for possible mutations of TMEM115 will be of interest.

## MATERIALS AND METHODS

### Cell lines

The HeLa-GalT-GFP stable cell line was a gift from Jack Rohrer (Friedrich Miescher Institut, Basel, Switzerland). HeLa-manII-GFP was a gift from Frederick Bard (Institute of Molecular and Cell Biology, Singapore). All other cell lines were obtained from American Type Culture Collection (Manassas, VA, USA).

### Antibodies and other materials

Antibodies against the following proteins were from the following sources: actin and Myc (Santa Cruz Biotechnology); HA (Roche); GM130 and CD8 (BD); mouse TMEM115 (Abnova); TMEM115, TGN46, FLAG and FLAG-conjugated agarose beads (Sigma-Aldrich); β-COP (Thermo Scientific); Golgin97 and COG3 were in-house antibodies (Loh and Hong, 2004; Lu et al., 2004). Fluorochrome-conjugated secondary antibodies were from Invitrogen and horseradish peroxidase (HRP)-conjugated secondary antibodies were from Jackson ImmunoResearch Laboratories, Inc. Brefeldin A was from Epicentre Biotechnologies. Digitonin and agarose-bound lectins were purchased from Sigma-Aldrich; fluorescent-dye-conjugated lectins were from Vector Laboratories. The TMEM115 human cDNA ORF (accession number NM_007024) was obtained from OriGene USA. GFP–ERGIC53 plasmid was a kind gift from Hans-Peter Hauri (University of Basel, Switzerland).

### Transfection and gene silencing

All plasmid transfections were performed using Effectene. All siRNA duplexes were of the ON-TARGET*plus* SMARTpool type obtained from Dharmacon RNAi Technologies. siRNA duplexes were transfected into cells using RNAiMAX^TM^ transfection reagent according to manufacturer's protocol.

### Immunofluorescence microscopy

Cells grown on coverslips were washed twice with PBSCM (PBS supplemented with 1 mM CaCl_2_ and 1 mM MgCl_2_) and then fixed in PBSCM containing 3% paraformaldehyde for 20 minutes. Fixed cells were washed five times at 5-minute intervals with PBSCM. The cells were permeabilized with 0.1% saponin (Sigma) in PBSCM for 15 minutes. Cells were then immunolabeled with appropriate primary antibodies diluted in fluorescence dilution buffer (PBSCM with 5% FBS and 2% BSA) for 1 hour at room temperature. The coverslips were then washed five times at 5-minute intervals with 0.1% saponin in PBSCM. Cells were subsequently incubated with secondary antibodies diluted in FDB for 1 hour at room temperature. The coverslips were washed five times at 5-minute intervals with 0.1% saponin PBSCM and then rinsed twice with PBSCM. The cells were then mounted on microscopic slides with Vectashield mounting medium (Vector Laboratories). Confocal microscopy was performed with Zeiss AxioplanII microscope (Oberkochen, Germany) equipped with a Zeiss confocal scanning optics.

### Surface biotinylation and lectin binding

Cells were biotinylated twice (15–20 minutes each) on ice with 0.5 mg/ml EZLink® sulfo-NHS-biotin (sulfo-N-hydroxysuccinimidobiotin, Pierce). The reaction was stopped by washing the cells four times (10 minutes each) with 50 mM NH4Cl at 4°C and then rinsing twice (10 minutes each) with ice-cold PBSCM. The biotinylated cells were scraped off the plate and then lysed in lysis buffer (25 mM Tris-HCl pH 7.5, 250 mM NaCl, 5 mM EDTA, 1% Triton X-100, 1% BSA, 10% FBS, and 1 mM PMSF) at 4°C with agitation for 1 hour. The extracts were centrifuged at 16,000 ***g*** for 10 minutes at 4°C. The supernatants were then incubated with streptavidin-agarose (Pierce) at 4°C for 2 hours. After washing once with lysis buffer, three times with buffer A (25 mM Tris-HCl pH 7.5, 500 mM NaCl, 0.5% Triton X-100, and 1 mM PMSF), and three times with buffer B (10 mM Tris-HCl pH 7.5, 150 mM NaCl), the beads were then eluted by boiling for 5 minutes in 2× Laemmli sample buffer, without Coomassie Blue and DTT. The eluted samples were then diluted in 4 ml lectin binding buffer (40 mM Tris-HCl pH 7.5, 150 mM NaCl, 1 mM CaCl_2_, 1 mM MgCl_2_, and 1 mM MnCl_2_) and then incubated with lectin–agarose at 4°C for 2 hours. The beads were then washed extensively, boiled in 2× Laemmli sample buffer for 5 minutes and analyzed by SDS-PAGE and western blotting.

### Immunoprecipitation

Cells on tissue culture dishes were lysed in lysis buffer [50 mM Tris-HCl pH 7.5, 150 mM NaCl, 1% Triton X-100, 1 mM PMSF, complete EDTA-free protease inhibitors and protein phosphatase inhibitors (Roche Diagnostics)]. The lysate was incubated on ice for 30 minutes and cleared by centrifugation at 16,000 ***g*** for 30 minutes at 4°C. Immunoprecipitation was carried out at 4°C with 5 µg of antibody in the presence of either Protein A or Protein G Sepharose 4 Fast Flow (GE Healthcare) for 4 hours with rotation. The sepharose was then washed five times with cell lysis buffer and twice with cold PBS. Bound proteins were eluted with 2× Laemmli sample buffer, resolved by SDS-PAGE and transferred onto PVDF membrane (Millipore) for subsequent immunodetection.

### *In vitro* translation and binding experiments

The TNT T7 Quick Coupled Transcription/Translation System (Promega) and the 1-Step Human Coupled *in vitro* Protein Expression Kit (Thermo Scientific) were used for *in vitro* translation according to the manufacturer's protocol. The translated products were combined in cell lysis buffer and subjected to FLAG immunoprecipitation. The precipitates were then washed five times with cell lysis buffer containing either 150 mM or 500 mM NaCl and twice with cold PBS. Bound proteins were eluted with 2× Laemmli sample buffer, resolved by SDS-PAGE and transferred onto PVDF membrane (Millipore) for subsequent immunodetection.

## Supplementary Material

Supplementary Material
