# Supplementary material for: TMEM115 is an integral membrane protein of the Golgi complex involved in retrograde transport
Source: J Cell Sci. 2014 Jul 1;127(13):2825–39. doi: 10.1242/jcs.136754 (PMC4077589; doi:10.1242/jcs.136754)
Supplement: Supplementary Material [file supp_127_13_2825__index.html]

TMEM115 is an integral membrane protein of the Golgi complex involved in retrograde transport — Supplementary Material 

# TMEM115 is an integral membrane protein of the Golgi complex involved in retrograde transport

## JCS136754 Supplementary Material

**Files in this Data Supplement:**

- **Supplementary Material**
