# Supplementary material for: TMEM115 is an integral membrane protein of the Golgi complex involved in retrograde transport
Source: J Cell Sci. 2014 Jul 1;127(13):2825–39. doi: 10.1242/jcs.136754 (PMC4077589; doi:10.1242/jcs.136754)

Fig. S1. (A) Flow chart elucidating the process for the identification of new candidate Golgi membrane proteins. (B) Hydrophobicity analysis of TMEM115 protein sequence using TMpred. (C) Alignment of amino acid sequences of TMEM115 from different organisms [human, mouse (m), frog (fr), zebrafish (zf), fly (f), worm (wo) and yeast (ye)]. The 4 hydrophobic regions were indicated by boxes whereas the antigenic epitope recognized by Atlas antibody (HPA015497) is indicated below the alignment. The predicted transmembrane topology is also shown.

Fig. S2. (A) Immuno-gold labelling of TMEM115. Immuno-electron analysis shows that TMEM115 is localised to the Golgi apparatus and is distributed towards the outer periphery of the Golgi. (B) TMEM115 antibody cannot co-IP COG3 and β-COP. HEK293 cell lysates were subjected to immunoprecipitation with TMEM115 antibody and rabbit IgG control. β-COP and COG3 did not co-precipitate with TMEM115. As the antigenic epitiope used to generate the TMEM115 antibody was located at the C-terminal region (supplementary material Fig. S1C), it likely blocks the domain that is required for their interaction. (C) TMEM115 does not co-IP p230 and Golgin-84. TMEM115-FL-FLAG was transfected into HEK293 cells. Lysates derived from control cells (Ctrl, lanes 2 and 4) or transfected cells (FL, lanes 1 and 3) were subjected to FLAG immunoprecipitation. p230 is a Golgi peripheral protein (Kjer-Nielsen et al., 1999) while Golgin-84 is a single transmembrane Golgi integral protein (Bascom et al., 1999). Results from these additional negative controls indicate that the interactions of TMEM115 and the various interacting partners (ERGIC53, β-COP and COGs) are specific.

Fig. S3. (A) BFA-dependent Golgi disassembly is delayed in TMEM115 and mutants that shows Golgi localisation. C-terminally FLAG-tagged TMEM115 full-length and mutants were transfected into HeLa-GalT-GFP cells. Cells were then incubated with 5 ∀g/ml BFA at 37°C for 10 min before being fixed and processed for immunofluorescence analysis. TMEM115-FL showed an inhibition of BFA-induced Golgi disassembly. The strength of inhibition correlates with the level of expression of TMEM115. The higher the expression of exogenous TMEM115, the slower the rate of BFA induced Golgi redistribution (panel a and b, asterisks). The mutants that show localisation to the Golgi, namely TMEM115-NT229, TMEM115-A1, TMEM115-A2 and TMEM115-ΔCC (Fig. 6) also exhibited inhibitory effects on BFA induced Golgi redistribution (panels e, f, m, n, o, p, q and r). Bar: 10 µm. (B) Golgi compactness index of TMEM115 knockdown cells. The degree of Golgi compactness was quantified using $4\pi \times \mathrm{Area}/\Sigma \mathrm{Perimeter}^2$ (Bard et al., 2003). At least 20 cells were taken for each measurement. A higher compactness index indicates a greater compactness of the Golgi apparatus.

Fig. S4. TMEM115 expression in knockdown cells could be rescued using a siRNA resistant construct. (A) HeLa GalT-GFP cells were transfected with TMEM115 siRNA#1 or control siRNA. 48 hours after initial siRNA transfection, the cells were transfected once more with 1 ∀g of TMEM115-si#1R-FLAG. 72 hours after initial siRNA transfection, the cells were treated with 5 ∀g/ml BFA at 37°C for either 0 or 10 min. The cells were then fixed and examined by immunofluorescence microscopy. The percentage of cells with Golgi retention was also shown. n: number of cells counted. Bar: 10 µm. (B) Cell lysates were obtained from cells as described in (A) and subjected to Western Blot analysis.

Supplementary Figure 1

A

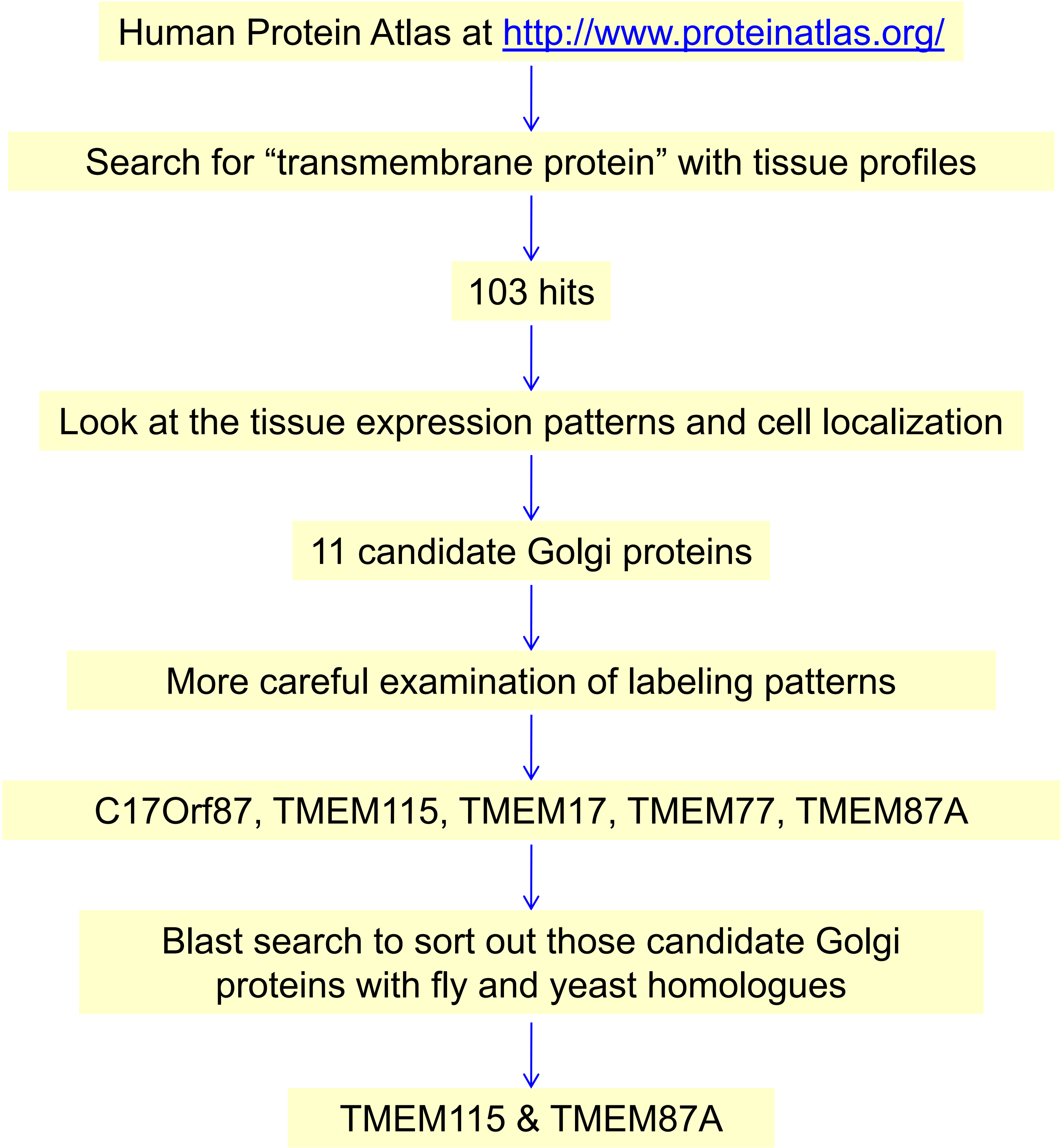

Supplementary Figure 1 Cont'd

B

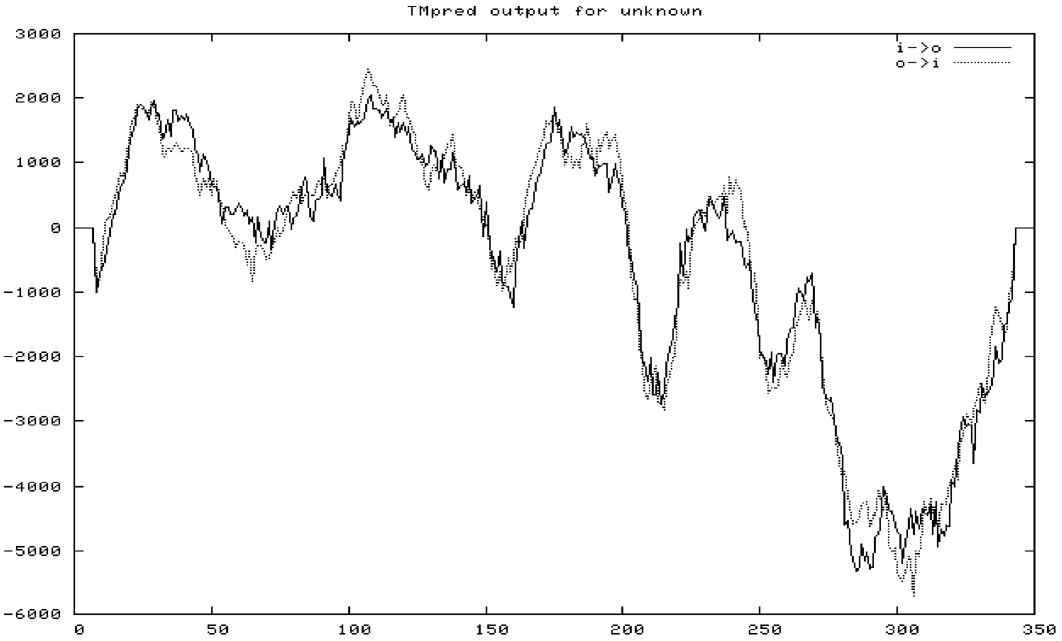

C

```
TMEM115.pr : -------MQRALPGARQHLGAILASASVVVKALCAAVLFLYLLSFAVDTG--CLAVTPGYLFPPN-------FWIWTLAT----HGLMEQHVWDVAISLTTVVVAGRLLEPLWG- :  94
TMEM115_(m : -------MQRALPGARQHLGAILASASVVVKALCAVVLFLYLLSFAVDTG--CLAVTPGYLFPPN-------FWIWTLAT----HGLMEQHVWDVAISLATVVVAGRLLEPLWG- :  94
TMEM115(fr : -------MLRSIHVGR--LVSVLSGSSVLVKCLWGAVLLLYLLSFWINTMTTALAVTPGLLLPTN-------LRIWTLAT----HGLLELNFCHVLCNLLLTLAAGRCLEPLWG- :  94
TMEM115(zf : -------MNRYLPVARQHFLSALASTSVVVKSICATVILLYLLSWAANTPY-LLGVTPGFLFPPN-------FWIWTPLT----HAVVEQHVFGMAVNIATVMVAGRLLEPLWG- :  95
TMEM115_(f : ---MSAQLARNWPYIWQQLNALLHNTSPVITLICVVTTFGYLLSFSETAIL-LLSVTPGYILPNG------KFWIWTAFT----FCFIELHWWEVAVDVVTVGLCGKMLEPLWG- : 100
TMEM115(wo : --MGVKAVLGDLQIVVERHSDVLRNNALPFRFLVVLRIIGYIISYLHFAR--LLLWLHPYQIAS--------IELWRIAT----STFCGHNVLDVLWTVWCLHFGTNLVRLNNT- :  98
TMEM115(ye : MQYSSRFLELNIPDSFLNINKIPDATKFITVTYICLTATLFCIRRSLYNKLVLEDPNLDYNLITSPLLQMVPSQIWRYPTSLVLSNFIDTKAWKVVVNLLNLIIGGSFIERNWNS : 115

TMEM115.pr : ALELLIFFSVVNVSVGLLGAFAYLLTYMASFNLVYLFTVRIHGALGFLGGVLVALKQTMGDCVVLRV-------PQVRVSVMPMLLLALLLLLRLATLLQSPA-LASYGFGLLSS : 201
TMEM115_(m : ALELLIFFSVVNVSVGLLGALAYLLTYMASFNLVYLFTIRIHGALGFLGGVLVALKQTMGDCVVLRV-------PQVRVSVVPMLLLALLLLLRLATLLQSPA-LASYGFGLLSS : 201
TMEM115(fr : APELLLFYGVVSVAVGILGSLVFLMAYAAAPHSYYLFATHIHGFSAFAGAVLVAHKQIAGDGQIE---------SKWWMQALPQLVLLLVMVTSLAGLIPSQM-FVGYSVGMLSG : 199
TMEM115(zf : ALELLIFFAVVNVAAGLLSGLSYLFTYAATFDLDYLFAVRVYGAPAFLGGVLVALKQTAGDTTVLRV-------PQVRLKAAPALALLAIAVLRLAGLLDTSAPLAACGYGALSG : 203
TMEM115 (f : QLEMFKFFALSNFGVSLLTTVYYLFYYMVTKNPTILFEVHIHGLAGYVAGICVAVRQIMPDHLIFKTRY-----GRLTNRNVPLTVLIMAIILWAIGLLDGTY-PAMFASGSLVS : 209
TMEM115(wo : NESLLKLYAITQGVTTFVIVVFAYLTYILFDSIKFFYIEPLVGMTPICASVMVLMKQFLPDTIVLATPL-----GRIKYAHLPFLAIFVSFILALTKFTYFVS-FLQITIGVQVS : 207
TMEM115(ye : SKEMFKFIIVLGSLTNVLIIMLTLLVSFFSN--KVRLDIPLDGNYTILIGFPIIYRQLLPETTIIHLKTPQFLAKNFRFKLLPIFVMFTMTVTQIIWFHHFAQ-LFSIWVTFFAS : 227

                                                    In
TMEM115.pr : WVYLRFYQRHSRGR-----------------GDMADHFAFATFFPEILQPVVGLLANLVHSLLVKVKICQK--TVKRYDVGAPSS--ITISLPGTD--PQDAERRRQLALKALNE : 293
TMEM115_(m : WVYLRFYQRHSRGR-----------------GDMADHFAFATFFPEILQPVVGLLANLVHGLLVKVKICQK--TVKRYDVGAPSS--ITISLPGTD--PQDAERRRQLALKALNE : 293
TMEM115(fr : WVYLRFYQRHSRGR-----------------GDMSDHFSFASFFPGPVQPAAALLGKVTHAALVKLHLCSQ--AVRRYDVGAPSS--ITISLPGTD--PQDAERRRQLALKALNE : 291
TMEM115(zf : WVYLRFYQRHSRGR-----------------GDMSDHFAFASFFPEALQPAVGFAAGLVHAALVKIKICRK--MVKRYDVGAPSS--ITISLPGTD--PQDAERRRQLALKALNE : 295
TMEM115_(f : WIYLRFYQHHPNGR-----------------GDSSESFTFVSFFPNVSQPFISVLVNPIYNCCLRAGVVKTPTPLRTISTASLTS--VSVQMPGVD--PHDIERRRQIALKALSE : 303
TMEM115(wo : WTYLRFYKPHETDEI---------------YGDGSEHFTWASLFPSRTQLFFTLIGKVCFRTLARMGVCKR--QVRHVDLHSLQSGSVGINLPALENSAKDSERRRQKALKELND : 305
TMEM115(ye : WSYLRFFQKLAPLNCPSLPTTNSQGGQEILVGDASDTFQLIYFFPDLIKPILRPIFNFIYNVVVKFKVIK--PFHDIDIDIGNT---IAESRGAKKIMTVEERRRQLALQVLEE : 337

                                 HPA015497
TMEM115.pr : RLKRVEDQ------SIWPSMDD--DEEESGAKVDSPLPSDK-------------------APTPPGKGAAP--------------------------------------------- : 337
TMEM115_(m : RLKRVEDQ------SAWPSMDD--DEEEAGAKTDSPLPLEE-------------------ASTPPGKVTVP--------------------------------------------- : 337
TMEM115(fr : RLKKVEDQ------VSWPNMEEE-EEEEDGNEIDSSFHSG--------------------DTPGDEPSIAQ--------------------------------------------- : 335
TMEM115(zf : RLKRVEDQ------SAWPSMEDEEDDEDEEVRTDTPLLSSR-------------------ETSQPAPSTTQNP------------------------------------------- : 343
TMEM115_(f : RLKATDSSRHAQLPKSFPQQQQLQHHHHQHAPHQQQKHHSHGTGHSHSHGAGHSHSHGAGHSHGPGPAQMPQPDFLKSTGSASQLPITTSRAEPRMISTMSPIAIPMPAPPPKEG : 418
TMEM115(wo : RLNKTRTA----EVASYGNWDDGDDDEPTEVTVVAPPPVS-------------------NVHSSKPAMDK--------------------------------------------- : 352
TMEM115(ye : RMVNP----------------------------------------------------------------------------------------------------------------- : 342

TMEM115.pr : ---------------ESSLITFEAAPPTL--- : 351
TMEM115_(m : ---------------ESSLITLETAPLL---- : 350
TMEM115(fr : --------------------SYEAAPKV---- : 343
TMEM115(zf : -----------TGQQESSIISFEDAPTHS--- : 361
TMEM115_(f : NVPGQGAESSSSGGVEATLINLDDVPTTSSMA : 450
TMEM115(wo : ----------------------ESMA------ : 356
TMEM115(ye : -------------------------------- :   -
```

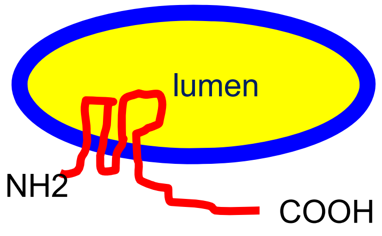

Predicted topology

Supplementary Figure 2

A

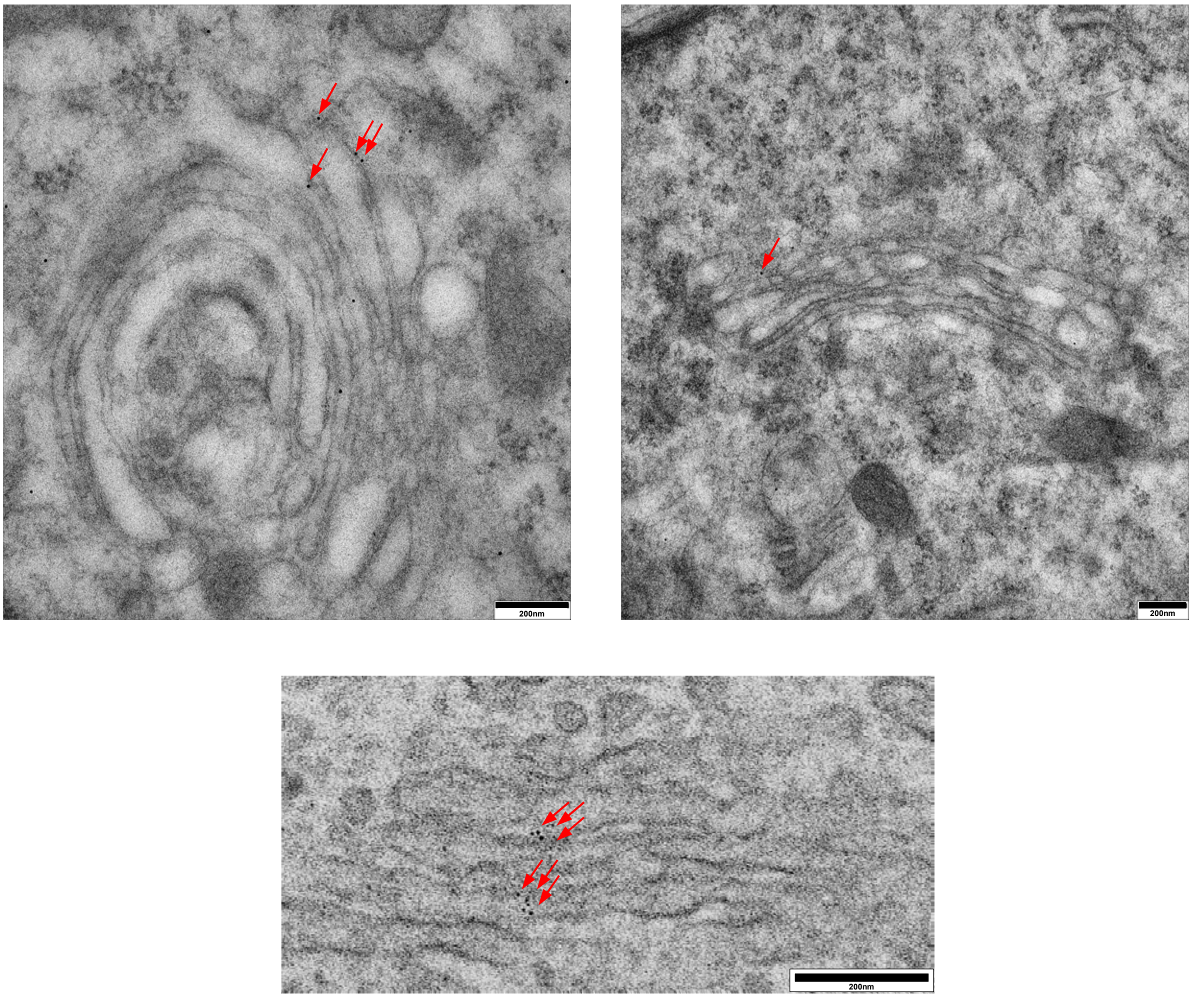

B

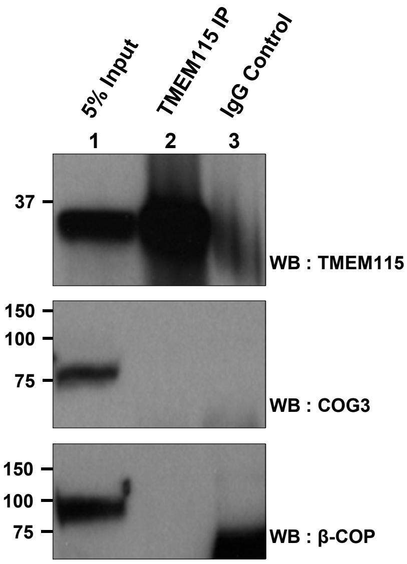

C

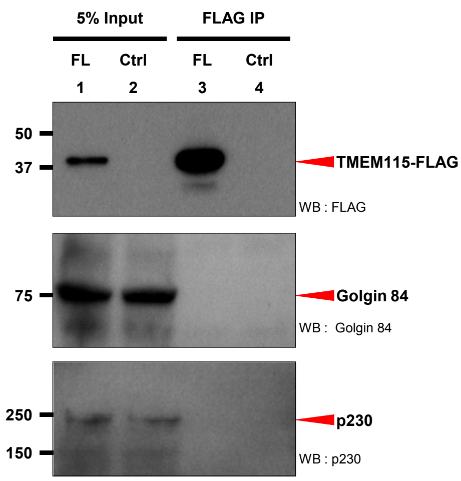

Supplementary Figure 3

A

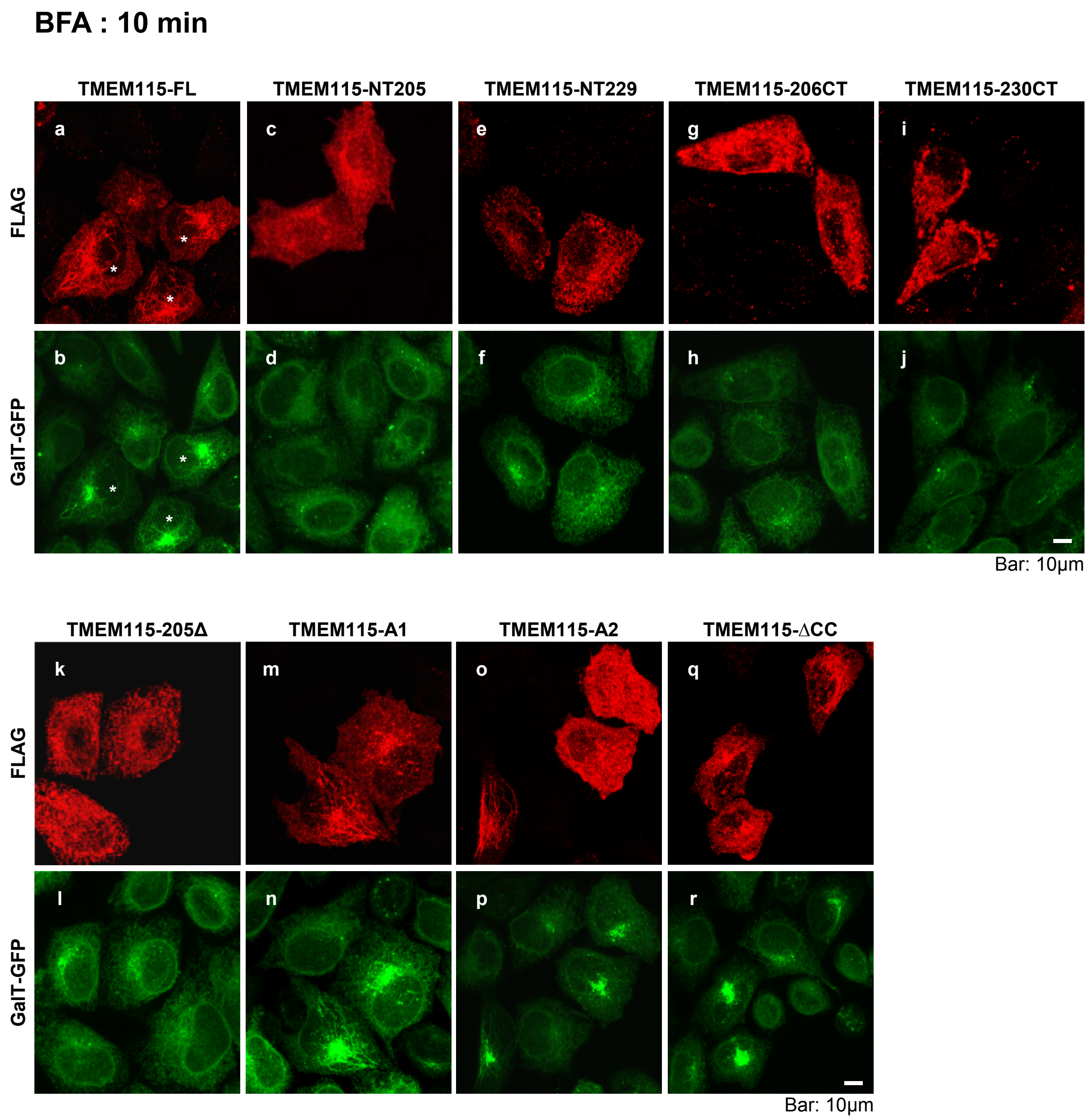

B

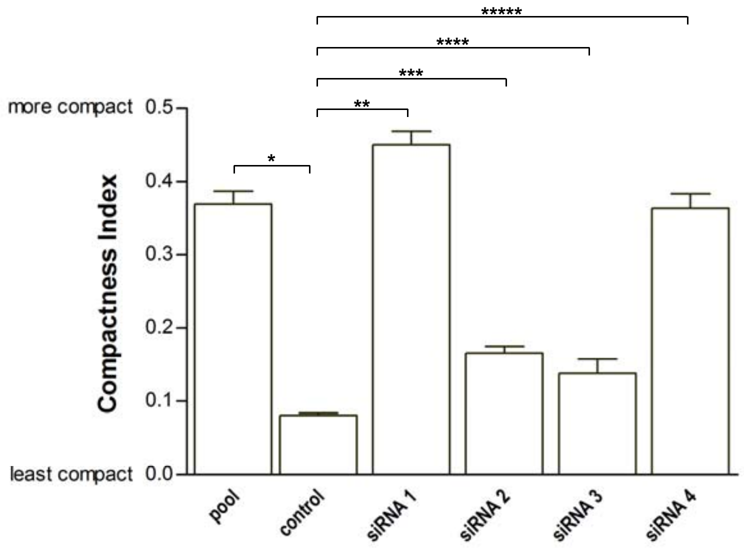

Supplementary Figure 4

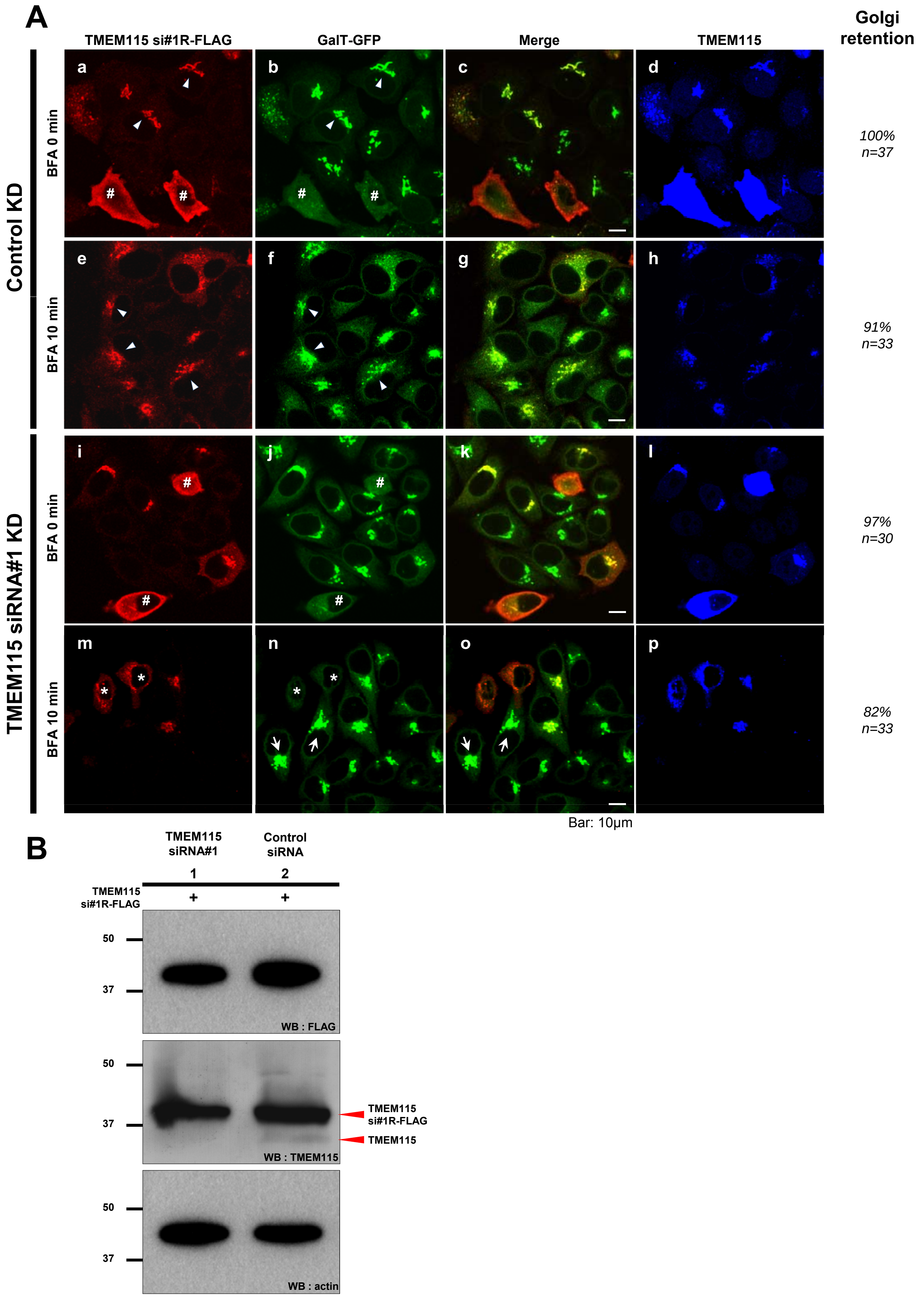

Supplement: Supplementary Material [file supp_127.13.2825_JCS136754.pdf]
